# Hydrogen sulfide attenuates intracellular oxidative stress via repressing glycolate oxidase activities in *Arabidopsis thaliana*

**DOI:** 10.1186/s12870-022-03490-3

**Published:** 2022-03-05

**Authors:** Lijuan Wang, Xiujie Mu, Xi Chen, Yi Han

**Affiliations:** 1 National Engineering Laboratory of Crop Stress Resistance Breeding, School of Life Sciences, Anhui Agricultural University, Hefei, 230036 China; 2 School of Food and Biological Engineering, Hefei University of Technology, Hefei, 230009 China; 3 School of Agronomy and Horticulture, Jiangsu Vocational College of Agriculture and Forest, Jurong, 212400 China

**Keywords:** Cell death, Hydrogen sulfide, Glycolate oxidase, H_2_O_2_, Oxidative stress, Salicylic acid

## Abstract

**Background:**

Hydrogen sulfide (H_2_S) has been proposed to exert anti-oxidative effect under many environmental stresses; however, how it influences oxidative stress remains largely unclear.

**Results:**

Here, we assessed the effects of H_2_S on oxidative stress responses such as salicylic acid (SA)-dependent cell death, which triggered by increased H_2_O_2_ availability in *Arabidopsis thaliana* catalase-deficient mutants *cat2* displaying around 20% wild-type catalase activity. H_2_S generation and its producing enzyme _L_-cysteine desulfhydrase (LCD/DES) were found to transient increase in response to intracellular oxidative stress. Although introducing the mutation of *des1*, an important LCD, into the *cat2* background produced little effect, H_2_S fumigation not only rescued the cell death phenotype of *cat2* plant, but also attenuated SA accumulation and oxidation of the glutathione pool. Unexpectedly, the activities of major components of ascorbate–glutathione pathway were less affected by the presence of H_2_S treatment, but decreased glycolate oxidase (GOX) in combination with accumulation of glycolate implied H_2_S treatment impacts the cellular redox homeostasis by repressing the GOX-catalyzed reaction likely via altering the major *GOX* transcript levels.

**Conclusions:**

Our findings reveal a link between H_2_S and peroxisomal H_2_O_2_ production that has implications for the understanding of the multifaceted roles of H_2_S in the regulation of oxidative stress responses.

**Supplementary Information:**

The online version contains supplementary material available at 10.1186/s12870-022-03490-3.

## Background

Abiotic stresses, such as drought, salinity, or heavy metal, are major constraint to productivity of all major crops worldwide. These environmental stresses influence plant yield and quality by affecting various cellular and whole-plant processes particularly through either reducing photosynthesis or the availability of water for basic cellular functions [1]. Moreover, these adverse factors can trigger rapid changes in the production and scavenging of reactive oxygen species (ROS) as its-associated oxidative stress has been assumed to be involved in these processes. For instance, hydrogen peroxide (H_2_O_2_) could affect several primary metabolic pathways such as carbon metabolism, glycolysis/gluconeogenesis, and amino acid biosynthesis presumably through the cysteine oxidation at specific proteins [2]. Stress-triggered ROS accumulation is mainly from mitochondrial respiration, photosynthesis in chloroplasts, peroxisome-localized photorespiration, and by apoplastic NADPH oxidases [3].

Other than its toxicity, H_2_S has been gradually accepted as a key messenger involved in a vast number of physiologically important processes in photosynthetic organisms, such as stress resistance, autophagy, stomatal movement [4–7]. The plant itself can generate H_2_S in different subcellular sites including cytosol, chloroplasts, mitochondria, and nuclei [8–13]. When plants are exposed to these adverse environmental cues, the production of H_2_S increases through enzymatic reactions mainly including the activation of sulfur assimilation pathway or the cysteine-degrading enzymes such as _D_-cysteine desulfhydrase (DCD) and _L_-cysteine desulfhydrase (LCD/DES) [6], Other factors and/or pathways such as β-cyanoalanine synthase and 3-mercaptopyruvate sulfurtransferases are also involved in H_2_S biogenesis [13–15]. Furthermore, due to its nucleophilic properties, H_2_S is capable of reacting with different ROS, thereby potentially severing as an antioxidant involving stress regulation [16]. However, other metabolites such as ascorbate and glutathione that are present within mM range at much higher concentrations than H_2_S [17], might be expected to be more effective in the removal of excess ROS. Instead of this, H_2_S could activate the transcriptional and/or translational levels of antioxidant enzymes and key biosynthetic enzymes of their substrates, to enhance ROS-processing capacity under many stress conditions. Indeed, numerous studies found that the major components including ascorbate peroxidase (APX), dehydroascorbate reductase (DHAR), glutathione reductase (GR) in ascorbate–glutathione pathway could be key regulatory nodes by H_2_S [18–20]. Treatment with H_2_S into several plant species under certain stress conditions was also observed to activate the expressions of L-galactose dehydrogenase, glutamate cysteine ligase and glutathione synthetase for ascorbate and glutathione synthesis, respectively [20–23]. As well as the effects on transcriptional regulation, H_2_S is proposed to be involved in generating cysteine persulfides on proteins, a process termed persulfidation which may lead to the alteration of protein functions. For example, H_2_S can drive persulfidation of certain antioxidant enzymes such as APX and the other peroxidase to potentiate their scavenging activities of ROS [19, 24]. Although it has been established that H_2_S-controlled redox balance could operate via up-regulation of antioxidant defense enzymes at the multiple levels, its effects on ROS biosynthesis in higher plants is much less understood except for the case studies that DES1-derived H_2_S participates in guard cell ABA signaling through modulating NADPH oxidase activity [25, 26].

In photosynthetic organisms, oxidative stress triggered by enhanced accumulation of ROS is common to environmental stress responses. Till now, studies on investigating the interactions between H_2_S and oxidative stress was only based on the analyses of effects of H_2_S on abiotic stress-triggered indirectly ROS accumulation and redox imbalance. In view of its importance in the context of oxidative stress, the aim of this work was to study the role of H_2_S in the regulation of oxidative responses by utilizing the *catalase2* (*cat2*) knockout mutants to directly induce stress responses such as salicylic acid (SA)-dependent cell death [27, 28], where H_2_O_2_ is produced in peroxisomes from O_2_ by the reaction of glycolate oxidase (GOX)-catalyzed glycolate oxidation during photorespiration.

## Results

### Effects of intracellular oxidative stress on H_2_S production and LCD activity

**Fig. 1:**
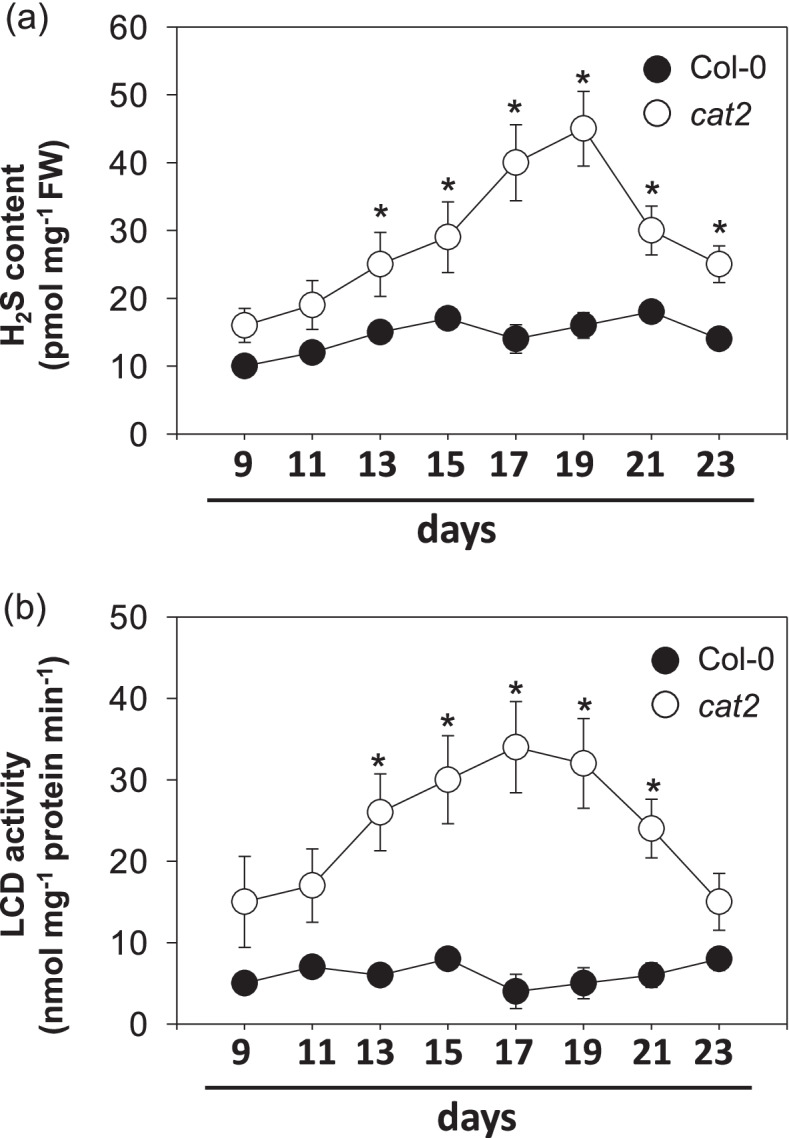
Time-course analysis of (**a**) H2S production and (**b**) LCD activity in Col-0 and cat2 seedlings. Time indicates days after seeds sowing. All values are means ± SE of three biological replicates. Asterisks indicate significant differences between cat2 and Col-0 at *P* < 0.05 at the same time point

Because studies of T-DNA insertion knockouts confirm CAT2 encodes the major leaf catalase isoform [29]. Increased H_2_O_2_ availability due to catalase deficiency in *cat2* knockout mutants led to the activation of intracellular oxidative stress responses mainly including the induction of SA-dependent cell death [27, 28]. Thus, we first assess the temporal profile of H_2_S production in response to increased H_2_O_2_ availability in *cat2* knockouts. H_2_S generation showed a marked transient increase in *cat2* mutants (Fig. 1a), with the initial increase correlating with the onset of lesions, which begin to be visible after about 14–15 days of growth [30]. During the same period of growth, isochorismate synthase-dependent SA accumulation gradually increased [28, 31]. Moreover, the extractable activities of LCD, a major H_2_S-generating component in *Arabidopsis*, gradually increased alongside H_2_S production in *cat2* mutants (Fig. 1b). These results demonstrate that the dynamic changes in *cat2*-impacted H_2_S levels are similar as *cat2*-triggered SA responses. A specific question we sought to answer was: Whether H_2_S is related to the H_2_O_2_-triggered SA pathway?

### DES1-derived H_2_S appears to be dispensable for oxidative stress responses

**Fig. 2:**
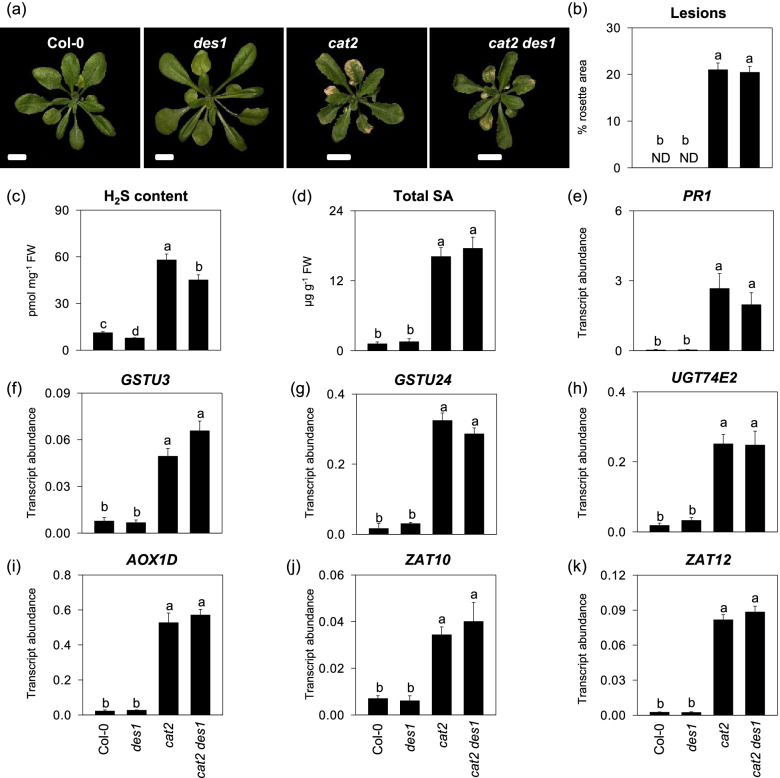
Influence of des1 mutation on cat2-induced phenotype, SA accumulation and associated gene expression, and ROSdependent genes. **a** Representative phenotypic image of twenty-day-old given plant genotypes. Scale Bars indicate 1 cm. **b** Lesion quantification in the different genotypes as a percentage of the total rosette area. ND, not detected. **c** H2S production. **d** and **e**, total SA and PR1 transcript level. In (**f**) to (**k**), selected ROS-related genes in Col-0, des1, cat2, and cat2 des1 by qPCR. Rosette samples of each line were taken after 20 days of growth. Values are means ± SE of three biological replicates. Different letters indicate significant differences at *P* < 0.05 by multiple pairwise t-test comparisons at *P* < 0.05

The function of DES1 has been proposed in stress responses and disruption of *DES1* provokes a 30% decrease in the amount of endogenous H_2_S concentration [25, 32]. We next examined if DES1-produced H_2_S is involved in modulating stress responses by genetically crossing *cat2* with *des1* knockout mutants. Phenotypic analysis showed that the *des1* mutation failed to alter spontaneous cell death triggered by *cat2* (Fig. 2a and b)*,* although disrupted DES1 function significantly reduced *cat2*-indcued H_2_S production (Fig. 2c). Consistent with cell death phenotypes, SA levels and *PATHOGENESIS-RELATED GENE 1* (*PR1*) transcripts, indicators of SA-dependent pathways, in *cat2 des1* and *cat2* mutants were indistinguishable after twenty days of growth (Fig. 2d and e). These observations indicate that DES1-sourced H_2_S likely had little effect on oxidative stress responses. To further examine above, six H_2_O_2_-responsive genes including *GLUTATHIONE S-TRANSFERASE TAU 3* (*GSTU3*), *GLUTATHIONE S-TRANSFERASE TAU 24* (*GSTU24*), *URIDINE DIPHOSPHATE GLYCOSYLTRANSFERASE 74E2* (*UGT74E2*), *ALTERNATIVE OXIDASE 1D* (AOX1D), *ZINC-FINGER OF ARABIDOPSIS THALIANAA 10* (*ZAT10*) and *ZINC-FINGER OF ARABIDOPSIS THALIANA 12* (*ZAT12*) were selected [33]. All of them were markedly induced in *cat2* mutants [34], but their transcript levels from *cat2 des1* leaves were comparable to those from single *cat2* mutants (Fig. 2f to k). Nevertheless, increased H_2_O_2_ in the leaves of *cat2* is difficult to detect. As measured by several approaches in our previous studies, no difference in leaf H_2_O_2_ levels was observed between *cat2* and Col-0 [31, 34]. This observation is consistent with previous studies of catalase-deficient arabidopsis, tobacco and barley lines [28, 35, 36]. Engagement of other H_2_O_2_-metabolizing pathways in catalase deficient plants is evidenced by adjustments in antioxidant systems [27, 31]. To assess the impact of the *des1* mutation on the H_2_O_2_-antioxidant interaction. The extractable activities of major antioxidant enzymes in the ascorbate–glutathione pathway as well as CAT, were measured. effects on activities of ascorbate peroxidase (APX), dehydroascorbate reductase (DHAR) and glutathione reductase (GR) in *cat2 des1* were comparable to those observed in *cat2* (Supplemental Figure S1).

**Fig. 3:**
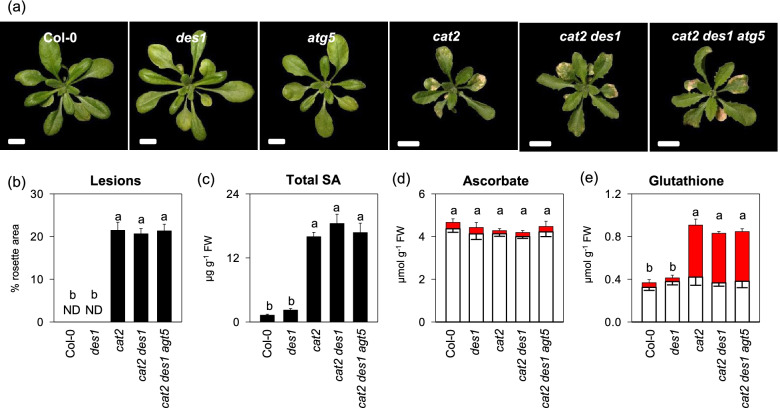
Analysis of phenotype, SA content and redox buffer in the given double and triple mutants. **a** representative image of twenty-day-old indicated genotypes. Scale Bars indicate 1 cm. **b** Lesion quantification in the different genotypes as a percentage of the total rosette area. ND, not detected. **c** Leaf total SA. **d** and **e** Leaf ascorbate and glutathione content. White bars, reduced form. Red bars, oxidized form. Samples of each line were taken after 20 days of growth. Values are means ± SE of three biological replicates. Different letters indicate significant differences by multiple pairwise t-test comparisons at *P* < 0.05

While autophagy plays crucial roles against oxidative stress, its pathway can be induced in the presence of *des1* mutation or oxidative stress conditions such as H_2_O_2_ treatment and nitrogen deprivation [32, 37, 38]. We therefore assess if autophagy contributes to oxidative stress responses by complementing the function of DES1. Because AUTOPHAGY 5 (ATG5) is a core component in autophagy [39]. We used *atg5* knockout mutants as a representative of autophagy-defective mutants and generated *cat2 des1 atg5* triple mutants for further experiments. As we unexpected, cell death and SA content were comparable in *cat2*, *cat2 des1* and *cat2 des1 atg5* mutants (Fig. 3a and b). Ascorbate and glutathione, both of which are key redox antioxidant buffers, were also determined. As reported previously, ascorbate is less affected in *cat2* than is glutathione [30]. No difference in ascorbate contents was observed in any of the mutants relative to Col-0 (Fig. 3d). By contrast, compared with wide-type Col-0 plants, total glutathione and its oxidized form (GSSG) substantially accumulated in *cat2*, However, *des1* and *des1 atg5* combined mutations in the *cat2* background produced little effect on glutathione state in comparison with the *cat2* single mutation (Fig. 3e).

### Effects of H_2_S exposure on SA-dependent cell death and redox status

**Fig. 4:**
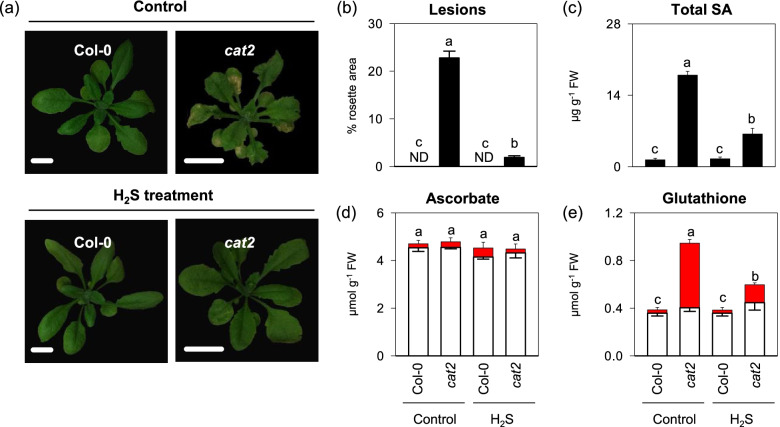
Effects of H2S exposure on phenotype, SA content and redox buffer in cat2 mutants. **a** representative image of twenty-day-old indicated genotypes. Scale Bars indicate 1 cm. **b** Lesion quantification in the different genotypes as a percentage of the total rosette area. ND, not detected. **c** Leaf total SA. **d** and **e** Leaf ascorbate and glutathione content. White bars, reduced form. Red bars, oxidized form. 16-day-old seedlings of Col-0 and cat2 were treated with gaseous H2S released from 0.5 mM NaHS solution under standard growth conditions for another 4 d. Values are means ± SE of three biological replicates. Different letters indicate significant differences at *P* < 0.05 by multiple pairwise t-test comparisons at *P* < 0.05

To further investigate the effect of the H_2_S on the H_2_O_2_-triggerecd oxidative responses, a H_2_S donor NaHS was employed for H_2_S fumigation treatment (See Materials and methods for details). As shown in Fig. 4a and b, H2S exposure did not impact leaf phenotype of wild-type background Col-0, but apparently attenuated H_2_O_2_-elcited lesion formation and SA accumulation in the *cat2* background (Fig. 4a to c), suggesting that this could be related to effects on enhanced antioxidant capacity or decreased H_2_O_2_ generation. As a result, the ascorbate status of H_2_S-treated Col-0 and *cat2* was similar to those of H_2_S-free control lines. No effect on ascorbate was observed (Fig. 4d)., The glutathione reduction state, which was above 90% in Col-0, was only 43% in *cat2*. Strikingly, glutathione in *cat2* was significantly less oxidized in the presence of H_2_S treatment, and this was accompanied by decreased total glutathione (Fig. 4e).

**Fig. 5:**
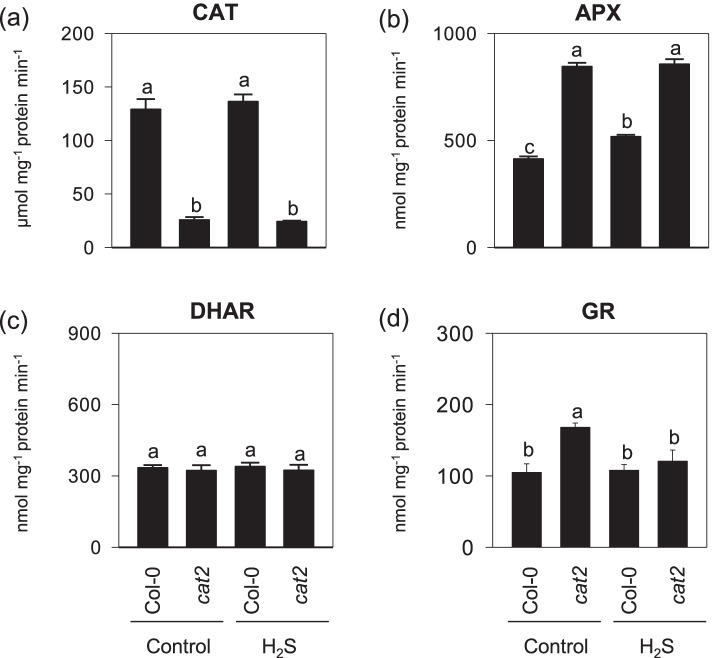
Effects of H2S exposure on major antioxidative enzyme in cat2 mutants. **a** CAT. **b** APX. **c**, DHAR. **d** GR. 16- day-old seedlings of Col-0 and cat2 were treated with gaseous H2S released from 0.5 mM NaHS solution under standard growth conditions for another 4 d. Values are means ± SE of three biological replicates. Different letters indicate significant differences at *P* < 0.05 by multiple pairwise t-test comparisons at *P* < 0.05

Furthermore, the activities of CAT, APX, GR, and DHAR, the enzymes linking glutathione pools [3], were measured (Fig. 5). As reported previously, the *cat2* mutant showed only about 20% of wild-type leaf catalase activity [27], and this was not affected by the H_2_S treatment (Fig. 5a). The activities of two other enzymes of the ascorbate–glutathione pathway, APX and GR, were increased in *cat2*, but H_2_S exposure did not promote or had little effect on either (Fig. 5b and d). Compared with Col-0, no increase in extractable DHAR activity was detected in all tested lines and treatments (Fig. 5c). Taken together, these results provide little evidence that exogenously applied H_2_S alleviated H_2_O_2_-triggered oxidative stress responses by enhancing the scavenging capacities of H_2_O_2_ related with ascorbate–glutathione pathway. Rather, the data indicate that H_2_S-fumiagted *cat2* plants showed a more reduced cellular redox environment.

**Fig. 6:**
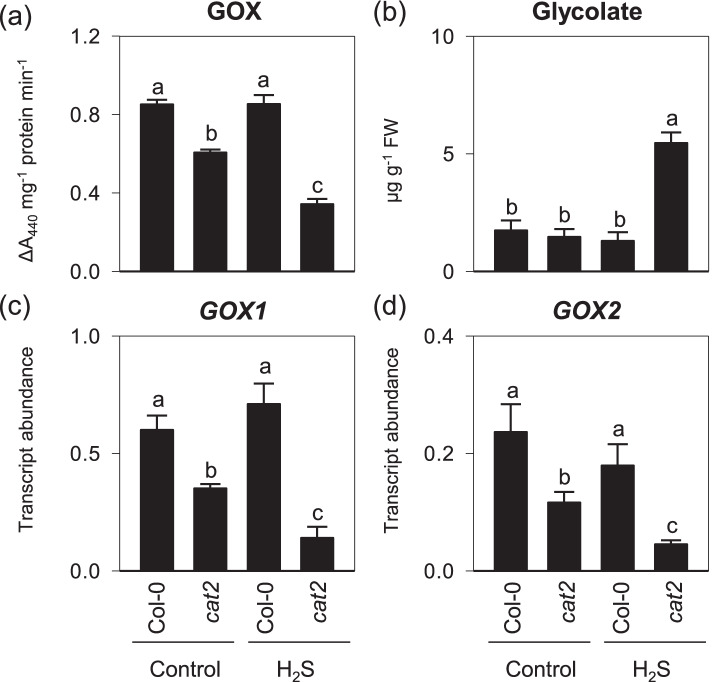
Effects of H2S exposure on photorespiratory H2O2 -producing enzyme and associated substrate in cat2 mutants. **a** and **b**, Extractable leaf GOX activity and glycolate content. **c** and **d**, GOX1 and GOX2 transcript levels. 16-day-old seedlings of Col-0 and cat2 were treated with gaseous H2S released from 0.5 mM NaHS solution under standard growth conditions for another 4 d. Values are means ± SE of three biological replicates. Different letters indicate significant differences by multiple pairwise t-test comparisons at *P* < 0.05

Because H_2_O_2_ generated from the reaction of glycolate oxidase (GOX)-catalyzed glycolate oxidation during photorespiration is metabolized by CAT. Decreased GOX activity thus might limit H_2_O_2_ accumulation. To examine whether the more reduced redox environment in the presence of H_2_S is due to the alteration of GOX activity, we compared the activities of GOX and the contents of its substrate glycolate between Col-0 and *cat2* in the absence or presence of H_2_S treatments. In agreement with our previous results and others [33, 34, 40], decreased GOX activity was observed in the control treatment of *cat2* leaves, but glycolate remained at Col-0 levels. H_2_S-fumigated *cat2* mutants displayed significantly lower GOX than did the control treatment of *cat2* mutants, accompanying by elevated levels of glycolate (Fig. 6a and b). Furthermore, the impaired GOX activity in H_2_S-treated *cat2* plants correlates with reduced transcript abundancies of the two major GOX isoforms *GOX1* and *GOX2* (Fig. 6c and d). These observations infer that H_2_S exposure regulated *cat2*-triggered oxidative stress responses likely via repressing the GOX-catalyzed reaction.

## Discussion

Our previous work demonstrated that increased H_2_O_2_ availability in the *cat2* mutants could increase the metabolism of cysteine and glutathione [31], both of which are essential for redox homeostasis and signaling [17]. Glutathione-dependent pathways were reported to be involved in oxidative stress responses such as the activation of SA and JA pathways downstream of H_2_O_2_ [30, 31, 34]. However, the functional actions of cysteine-related in the regulation of oxidative stress remains to be determined. H_2_S generation via cysteine-degrading reactions has been shown to be induced in response to various environmental stresses [6]. Hence, uncovering if and how H_2_S participates in stress responses has had more paid attention in recent years. In this study, H_2_S production, and the activities of LCD, an enzyme involved in cysteine degradation, were observed to transient increase in response to intracellular oxidative stress. DES1 is by far the best-characterized cysteine desulfhydrase, which confers plant stress acclimation including nitrogen starvation responses, senescence, stomatal closure, and immunity [32, 37, 41, 42]. Nevertheless, the *des1* mutation seems to have less apparent effect on oxidative stress responses in *cat2* (Fig. 2). Moreover, autophagy, which was regulated by DES1-derived H_2_S signaling [32], failed to affect above process (Fig. 3). It should be noted that the *des1* mutation led to only 30% and 22% reduction in endogenous sulfide in wild type and *cat2* background respectively, and identified very few proteins (47 proteins) with decreased levels of persulfidation relative to wild-type plants [32, 43], indicating a limited role of DES1 in H_2_S signaling. Alternatively, this could raise another possibility that other and/or unidentified factors/pathways promoting H_2_S generation may compensate for the loss of function of DES1 if they have redundant roles in the regulation of oxidative stress. Indeed, several candidate genes, encoding the cysteine desulfhydrase, have been implicated in enhancing resistance to many stresses such as heavy metal stress, salt, drought and cold stress, pathogen attack, and preventing ROS accumulation [44–48]. For instance, cytosolic O-acetylserine-(thiol) lyase A (OASTLA) and chloroplastic OASTLB have substantially higher LCD activities than the cytosolic DES1, and likely act as major cysteine desulfhydrases in Arabidopsis [44].

Regardless of which specific isoforms of cysteine desulfhydrases are involved, the exposure to gaseous H_2_S led to the alteration in oxidative stress. Unlike other studies that reported that H_2_S modulated oxidative stress through elevating antioxidant capacities especially for the components in the ascorbate–glutathione pathway [18–20], our results presented here revealed an additional role of H_2_S in regulating stress responses. The photorespiratory GOX-generating H_2_O_2_ accounts for about 70% of the total pool and is therefore the most crucial H_2_O_2_ source in photosynthesizing C3 leaves [49]. Glycolate accumulation in H_2_S-treated *cat2*, jointly with decreased GOX activities and increased reduction status of glutathione (Fig. 4), implies that the peroxisomal H_2_O_2_ production via the photorespiratory pathway is reduced. Therefore, this partially restricted photorespiratory activity conferred by H_2_S treatment might be sufficient to attenuate the SA-associated cell death of *cat2* mutants. Because H_2_S has been considered to regulate oxidative stress at the multiple levels [6]. Thus, such inhibitory effect could be likely linked to decreased transcript levels of the major forms of GOX (Fig. 6) [40], but we cannot exclude the possibility that the H_2_S-based post-translational modifications exert an effect on GOX activities. Both GOX1 and GOX2 have a single, and highly conserved cysteine residue that potentially could serve as a site for multiple redox modifications governing protein functionality under oxidative conditions. In line with above speculation, GOX1 has been implicated in undergoing glutathionylation [50]. Several lines of evidence showed that GOX from *Kalanchoe pinnata* and *Pisum sativum* were susceptible to nitrosation, which displayed decreased catalytic activity [51, 52]. Intriguingly, Both GOX isoforms were found to be targets of persulfidation observed in *Arabidopsis* [43], but whether such modification could repress GOX activity is, at least in vitro, difficulty to determine. Because the S in H_2_S is in the same reduced state as thiols and cannot react with protein cysteine residues to form persulfides unless an oxidant is added. However, the direct addition of H_2_S and H_2_O_2_, even at similar concentrations is less efficient and uncontrollable. Very recently, Ni et al. [53] developed an unique method to induce efficient persulfidation on proteins under physiologically relevant H_2_O_2_/H_2_S concentrations. This system can simultaneously produce H_2_S and H_2_O_2_ in a slow and controllable manner as this could be extensively applied to assess the effects of persulfidation in the future of H_2_S biological research.

## Methods

### Plant material and growth conditions

*Arabidopsis thaliana* wild-type Columbia-0 (Col-0) and homozygous knockout lines including *cat2* (SALK076998) [27], *atg5* (SAIL_129B07) [54] and *des1* (SALK_103855) [32]used in this study, were obtained from the Arabidopsis Biological Research Center (ABRC), Columbus, Ohio, USA. A triple mutant *cat2 des1 atg5* and a double mutant *cat2 des1* were generated for this study. Above mutant lines used in this study were verified by PCR. All specific PCR primers used for genotyping are listed in Supplemental Table S1. Seeds were incubated for 2 d at 4ºC and then sown in soil. Plants were grown in a controlled-environment growth chamber at a day/night regime of 16 h/8 h (light/dark), an irradiance of 150 μmol m^−2^ s^−1^ at leaf level, temperatures of 22 ºC day/20 ºC night, 65% humidity. Our study complied with relevant institutional, national, and international guidelines and legislation, and no specific permits were required to collect the plant samples. Samples were rapidly frozen in liquid nitrogen and stored at -80ºC until analysis. Unless otherwise stated, data are means SE of at least three independent samples from different plants.

### H_2_S fumigation treatment

The procedure of H_2_S fumigation was carried out as described in Wei et al. [42]. Solutions of sodium hydrosulfide (NaHS•3H_2_O) were used as a donor of H_2_S. In a sealed glass desiccator (volume 3 L), 16-day-old seedling lines were treated with gaseous H_2_S for 4 h per day, which was released from the 0.5 mM NaHS aqueous solution (200 mL). The aqueous solution of NaHS at 0 mM was set as the control. The NaHS solution and its control treatment were renewed each day and treated leaves were collected at designated time intervals for analyses.

### Determinations of H_2_S content and LCD activity

H_2_S quantification was performed as described by Singh et al. [55]. 0.5 g plant leaves were ground into fine powder with a mortar and pestle under liquid nitrogen and then homogenized in 1 ml of the following extraction buffer: 20 mM Tris–HCl buffer (pH 8.0), 10 mM EDTA, 20 mM Zn(OAc)_2_. The homogenate was centrifuged at 15,000 g for 15 min at 4ºC and 0.1 mL of the supernatant was used for the H_2_S quantification, in an assay mixture containing 1.88 mL extraction buffer and 0.02 mL of 20 mM 5,5’-dithiobis(2-nitrobenzoic acid). The reaction mixture was incubated at room temperature for 2 min and the absorbance was read at 412 nm. H2S was quantified based on a standard curve prepared with NaHS.

The activity of LCD was measured as described previously [11]. Freshly sampled or stored at -80 °C leaves (200 mg) were ground to a fine powder in liquid nitrogen, and the soluble proteins were extracted by adding 1 mL of 20 mM Tris–HCl (pH 8.0), and centrifuged at 15,000 g for 15 min at 4ºC. LCD activity was detected by monitoring the release of H2S from L-cysteine in the presence of dithiothreitol (DTT). The assay was performed in a total volume of 1 mL comprising 2.5 mM dithiothreitol (DTT), 0.8 mM L-cysteine, 100 mM Tris–HCl (pH 9.0), and 10 μg of protein solution. The reaction was initiated by the addition of L-cysteine after incubation for 15 min at 37 °C. and was terminated by the addition of 0.1 mL of 30 mM FeCl_3_ dissolved in 1.2 N HCl and 0.1 mL 20 mM N,N-dimethyl-p-phenylenediaminedihy drochloride dissolved in 7.2 N HCl. The formation of methylene blue was determined at 670 nm. LCD enzymatic activity was calculated using a standard curve of known concentrations of NaHS.

### Lesion quantification and SA assay

Percentage lesion area in *cat2* in the absence or presence of H_2_S, *cat2 des1* or *cat2 des1 atg5*, was quantified using IQmaterials software. Total SA was extracted as described in Langlois-Meurinne et al. [56]. SA was determined by HPLC-fluorescence according to the protocol of [31]. Identification and quantification was performed by comparison of peaks with SA standards.

### qPCR analysis

Total RNA was extracted with TRIzol reagent (Takara) following the manufacturer’s instructions. To avoid potential genomic DNA contamination, RNA was treated with RNase-free DNase I (Takara). RNA quality and concentration were determined by gel electrophoresis and estimated using a nanodrop spectrophotometer at 260 nm respectively. Reverse transcription and first-strand cDNA synthesis were performed using the All-in-One cDNA Synthesis SuperMix (Bimake). qPCR was performed according to Zhang et al. [34]. In all experiments, three biological replicates of each sample and three technical (PCR) replicates were performed. Primer sequences are listed in Supplemental Table S1.

### Ascorbate and glutathione assays

Oxidized and reduced forms of ascorbate and glutathione were quantified using the plate-reader method described by Queval and Noctor [57]. Leaf samples (100 mg) were ground in liquid nitrogen and then extracted into 1 mL of 0.2 N HCl. Before ascorbate and glutathione assays, the final pH of all samples was neutralized between 5 and 6. Ascorbate was measured by the absorbance at 265 nm that is specifically removed by ascorbate oxidase (AO). The reduced form was measured in untreated acid extract aliquots, while total ascorbate (dehydroascorbate + ascorbate) was measured following pre-incubation of aliquots with DTT to reduce dehydroascorbate to ascorbate. To assay ascorbate, the buffer contained 100 μL 0.2 M NaH_2_PO4 (pH 5.6), 40 μL extract and 55 μL H_2_O. The absorbance at 265 nm was read before and 5 min after adding 0.2 U of AO. Total ascorbate was measured after incubation of 100 μL neutralized extract with 140 μL 0.12 M NaH_2_PO4 (pH 7.5), 10 μL 25 mM DTT for 30 min at room temperature. Both of above assays were done in triplicate for each extract. According to a standard extinction coefficient of 14 mM^−1^ cm^−1^, absorbance changes in A_265_ were converted to quantities of ascorbate.

The method for glutathione measurement relies on GR-dependent reduction of DNTB by GSH monitored at 412 nm and is used to measure either total glutathione (GSH + GSSG) or GSSG. Specific assay of GSSG was done by pre-treatment of extract aliquots with 2 μL of 2-vinylpyridine (VPD), which efficiently complexes GSH. To measure total glutathione, the mixture contained 100 μL of 0.2 M NaH_2_PO4, 10 mM EDTA (pH 7.5), 10 μL 10 mM NADPH, 10 μL 12 mM DTNB, 10 μL of neutralized extract and 60 μL water. After shaking, the reaction was started by addition of 10 μL GR. GSSG measurement was based on the same principle except that extract aliquots were incubated with VPD for 30 min at room temperature and were centrifuged twice for 15 min at 4ºC before assay. The increase in absorbance at 412 nm was monitored for 5 min and all assays were done in triplicate for each extract. Rates were converted to quantities of glutathione using standard curves generated by assay of known concentration in the same plate.

### Measurements of glycolate oxidase activity and glycolate content

The glycolate oxidase activity was performed by monitoring the formation of colored O-dianisidine radical cation in the presence of sodium glycolate at 440 nm according to protocol detailed by Waszczak et al. [33]. Glycolate content was measured by liquid chromatography-tandem mass spectrometry (LC–MS/MS, Agilent), using 5% acetic acid extracts from leaves as described in Saji et al. [58].

### Measurements of antioxidant enzyme activities

Spectrophotometric assay to measure enzyme activities in the glutathione-ascorbate pathway was performed as described previously [59]. Briefly, freshly sampled or stored at -80 °C leaves or (150 mg) were homogenized with 1.5 mL extraction buffer containing 50 mg insoluble polyvinylpyrrolidine, 0.1 M phosphate buffer, 1 mM EDTA (pH7.5) and 1 mM ascorbate. Catalase was measured by the removal of H_2_O_2_ monitored at 240 nm, DHAR as GSH-dependent formation of ascorbate from DHA at 265 nm, APX as H_2_O_2_-dependent ascorbate oxidation at 290 nm, and GR as GSSG-dependent NADPH oxidation at 340 nm. Bradford’s method was adopted for measuring the protein concentration, and bovine serum albumin was used as standard [60].

### Statistical analysis

The significance of differences was determined by Student’s *t*-test. Calculations were performed on a minimum of three independent datasets, assuming two samples equal variance and a two-tailed distribution. For comparing multiple treatments, different letters indicate significant differences using multiple pairwise *t*-test comparisons at *P* < 0.05.

## Supplementary Information

**Additional file 1: Figure S1.** Effects of des1 mutation on major antioxidative enzyme in cat2 mutants. (a) CAT. (b), APX. (c), DHAR. (d), GR. Samples of each line were taken after 20 days of growth. Values are means ± SE of three biological replicates. Different letters indicate significant differences by multiple pairwise t-test comparisons at P < 0.05.

**Additional file 2: Table S1.** List of primers and restriction enzymes used in this study.

## Data Availability

The data presented in this study are available in the graphs provided in the manuscript. Plant materials used during the current study are available from the corresponding author upon reasonable request.
