# Supplementary material for: Hydrogen sulfide attenuates intracellular oxidative stress via repressing glycolate oxidase activities in Arabidopsis thaliana
Source: BMC Plant Biol. 2022 Mar 5;22:98. doi: 10.1186/s12870-022-03490-3 (PMC8897949; doi:10.1186/s12870-022-03490-3)
Supplement: Supplementary file 1 — Additional file 1: Figure S1. Effects of des1 mutation on major antioxidative enzyme in cat2 mutants. (a) CAT. (b), APX. (c), DHAR. (d), GR. Samples of each line were taken after 20 days of growth. Values are means ± SE of three biological replicates. Different letters indicate significant differences by multiple pairwise t-test comparisons at P < 0.05. [file 12870_2022_3490_MOESM1_ESM.pptx]

## Slide 1
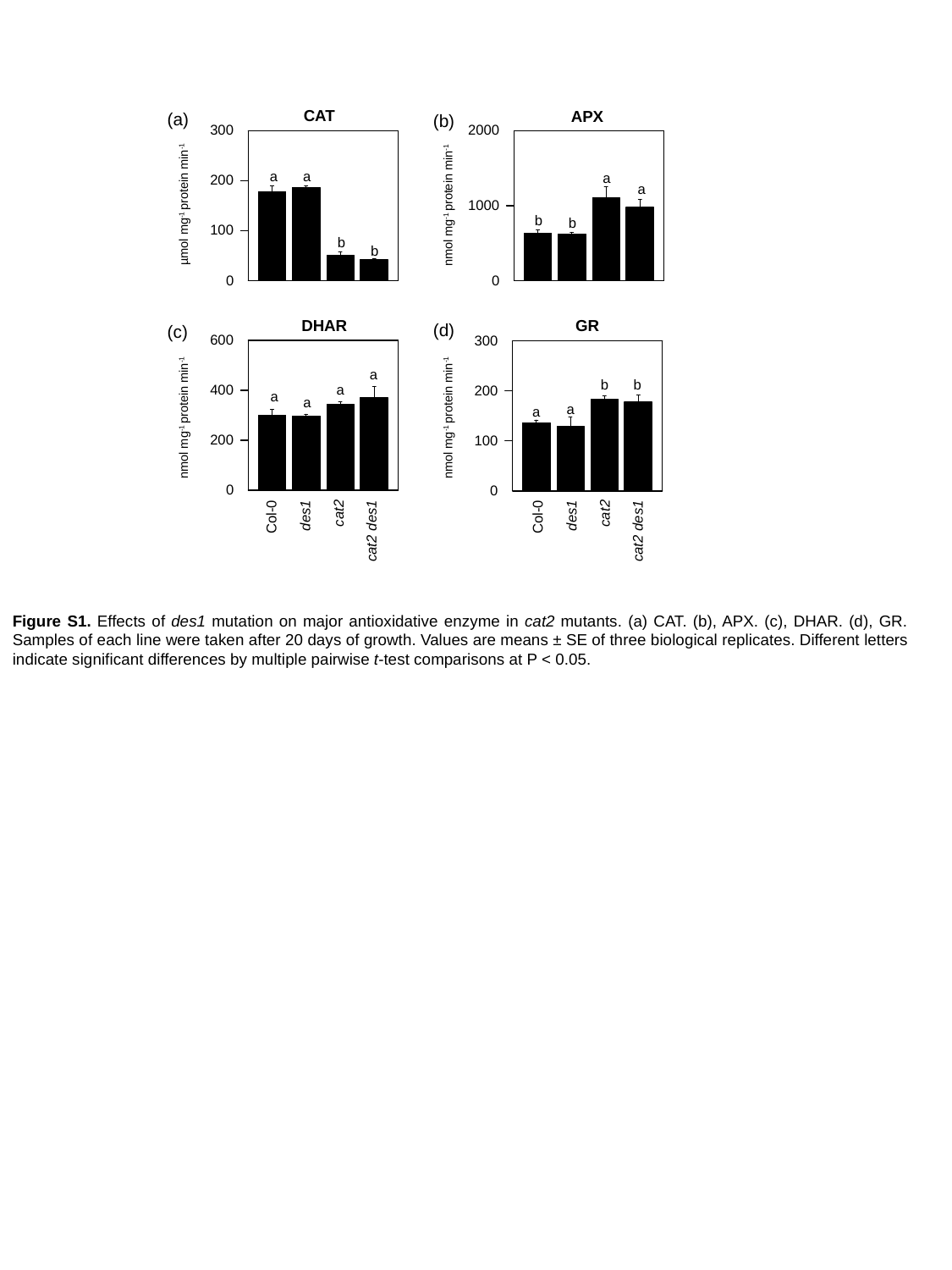

CAT
APX
(a)
(b)
a
a
a
a
µmol mg-1 protein min-1
nmol mg-1 protein min-1
b
b
b
b
DHAR
GR
(d)
(c)
a
b
b
a
a
a
a
a
nmol mg-1 protein min-1
nmol mg-1 protein min-1
cat2
des1
Col-0
cat2 des1
cat2
des1
Col-0
cat2 des1
Figure S1. Effects of des1 mutation on major antioxidative enzyme in cat2 mutants. (a) CAT. (b), APX. (c), DHAR. (d), GR. Samples of each line were taken after 20 days of growth. Values are means ± SE of three biological replicates. Different letters indicate significant differences by multiple pairwise t-test comparisons at P < 0.05.
