# Supplementary material for: Hydrogen sulfide attenuates intracellular oxidative stress via repressing glycolate oxidase activities in Arabidopsis thaliana
Source: BMC Plant Biol. 2022 Mar 5;22:98. doi: 10.1186/s12870-022-03490-3 (PMC8897949; doi:10.1186/s12870-022-03490-3)
Supplement: Supplementary file 2 — Additional file 2: Table S1. List of primers and restriction enzymes used in this study. [file 12870_2022_3490_MOESM2_ESM.docx]

**Supplemental Table S1.** List of primers and restriction enzymes used in this study.

| **N°** | **Oligonucleotide** | **Sequence** | **Technique** | |
| --- | --- | --- | --- | --- |
| 1 | ForCAT2 | 5’-CCCAGAGGTACCTCTTCTTCTCCCATG-3’ | Genotyping  Genotyping  Genotyping  Genotyping  Genotyping  Genotyping  Genotyping  Genotyping  RT-qPCR  RT-qPCR  RT-qPCR  RT-qPCR  RT-qPCR  RT-qPCR  RT-qPCR  RT-qPCR  RT-qPCR  RT-qPCR  RT-qPCR  RT-qPCR  RT-qPCR  RT-qPCR  RT-qPCR  RT-qPCR  RT-qPCR  RT-qPCR  RT-qPCR  RT-qPCR  RT-qPCR  RT-qPCR  RT-qPCR  RT-qPCR | |
| 2 | RevCAT2 | 5’-TCAGGGAACTTCATCCCATCGC-3’ |  |  |
| 3 | ForDES1 | 5’-GCGGTCTTTTGTCTCTTCTTC-3’ |  |  |
| 4 | RevDES1 | 5’-AGTAACCGTTCCACCAGTTCC-3’ |  |  |
| 5 | ForATG5 | 5’-GCTAATTGCACAAAGCTTACCTC-3’ |  |  |
| 6 | RevATG5 | 5’-TGATATGCCTAACATCGTCCAC-3 |  |  |
| 7 | SALK LB1 | 5’-TGGACCGCTTGCTGCAACTCTC-3’ |  |  |
| 8 | SAIL LB | 5’-GAAATGGATAAATAGCCTTGCTTCC-3 |  |  |
| 9 | ForYLS8 | 5’-AGAGCGTCTCGTCGTCATTC-3’ |  |  |
| 10 | RevYLS8 | 5’-GTCTCAGCAACAGACGCAAG-3’ |  |  |
| 11 | ForEF-1α | 5’-CTGGAGGTTTTGAGGCTGGTAT-3’ |  |  |
| 12 | RevEF-1α | 5’-CCAAGGGTGAAAGCAAGAAGA-3’ |  |  |
| 13 | ForACTIN2 | 5’-CTGTACGGTAACATTGTGCTCAG-3’ |  |  |
| 14 | RevACTIN2 | 5’-CCGATCCAGACACTGTACTTCC-3’ |  |  |
| 15 | ForPR1 | 5’-AGGCTAACTACAACTACGCTGCG-3’ |  |  |
| 16 | RevPR1 | 5’-GCTTCTCGTTCACATAATTCCCAC-3’ |  |  |
| 17 | ForGSTU3 | 5’-TGACGTTGCAATCGAAGAAG-3’ |  |  |
| 18 | RevGSTU3 | 5’-ACGACCATGTCCAAGAATCC-3’ |  |  |
| 19 | RorGSTU24 | 5’-AGACTTGGCCCGACAATAAC-3’ |  |  |
| 20 | RevGSTU24 | 5’-TCCTTCTCGCCGTAACATTC-3’ |  |  |
| 21 | ForUGT74E2 | 5’-GAATCGTCCTCATACCCGAAT-3’ |  |  |
| 22  23  24 | RevUGT74E2  ForZAT10  RevZAT10 | 5’-GCTTTGGACCCATTTCAACA-3’  5’-TCCCTGAATTCTCGATGGTC-3’  5’-TTTGACCGGAAAGTCAAACC-3’ |  |  |
| 25 | ForZAT12 | 5’-TCCAAGCCTTAGGAGGTCAC-3’ |  |  |
| 26 | RevZAT12 | 5’-ACAAAGCGTCGTTGTTAGGC-3’ |  |  |
| 27 | ForAOX1D | 5’-ACGTCATCTCCGGTAAGTGG-3’ |  |  |
| 28 | RevAOX1D | 5’-CCATGGCTGAAAACAATTCC-3’ |  |  |
| 29 | ForGOX1 | 5’-AAAGATGGCTCACCCTGATG-3’ |  |  |
| 30 | RevGOX1 | 5’-CTTCAACGCTGGAAGTAGCC-3’ |  |  |
| 31 | ForGOX2 | 5’-AGTGGCTCCAAACAATCACC-3’ |  |  |
| 32 | RevGOX2 | 5’-CGCTATCCTTGCATCCTCTC-3’ |  |  |
|  |  |  |  |  |
